# Membrane Surface Features of *Blastocystis* Subtypes

**DOI:** 10.3390/genes9080417

**Published:** 2018-08-17

**Authors:** John Anthony Yason, Kevin Shyong Wei Tan

**Affiliations:** Laboratory of Cellular and Molecular Parasitology, Department of Microbiology and Immunology, Yong Loo Lin School of Medicine, National University of Singapore, Singapore 119077, Singapore; mictank@nus.edu.sg

**Keywords:** *Blastocystis*, surface coat, subtypes

## Abstract

*Blastocystis* is a common intestinal protistan parasite with global distribution. *Blastocystis* is a species complex composed of several isolates with biological and morphological differences. The surface coats of *Blastocystis* from three different isolates representing three subtypes were analyzed using scanning electron microscopy. This structure contains carbohydrate components that are also present in surface glycoconjugates in other parasitic protozoa. Electron micrographs show variations in the surface coats from the three *Blastocystis* isolates. These differences could be associated with the differences in the pathogenic potential of *Blastocystis* subtypes. Apart from the surface coat, a plasma membrane-associated surface antigen has been described for *Blastocystis* ST7 and is associated with programmed cell death features of the parasite.

## 1. Introduction

*Blastocystis* is a eukaryotic unicellular organism commonly detected in the intestinal tract of humans and many animals [1,2]. There are one billion individuals estimated to be infected by *Blastocystis* worldwide [3]. The parasite is classified under stramenopiles, although it is an atypical member of this group. Stramenopiles usually possess flagella and surface tubular hairs, but *Blastocystis* have neither [4,5]. The life cycle of *Blastocystis* is initiated upon ingestion of cysts by the host. The parasite then subsequently excyst, develop into vacuolar forms, and colonize the large intestines. Some of the vacuolar forms undergo encystation, and the cysts that develop from this process are passed in the feces. These can then be transmitted to other human and animal hosts [5,6]. *Blastocystis* reproduce by binary fission, as observed from microscopic analyses of clinical and laboratory samples. Alternative modes of reproduction have been suggested [7], but have yet to be validated [2,5]. Infection with *Blastocystis* has been associated with several gastrointestinal symptoms, although most cases are asymptomatic. The pathogenic potential of *Blastocystis* thus still needs to be evaluated [2,3,5]. The parasite is composed of different subtypes (STs), based primarily on the organism’s small-sub unit ribosomal RNA (SSU-rRNA) gene sequences [8]. There are currently 17 STs in mammalian and avian hosts and STs 1–9 have been found in humans. These STs exhibit differences in morphology and host ranges, as well as drug susceptibility and induction of host immune responses [5,9,10]. *Blastocystis* ST1 and ST3 are detected in most surveys in human populations, while ST4 are found to also be common in Europe [11]. *Blastocystis* ST2, along with ST1 and ST3, are also detected frequently in South American surveys [12]. *Blastocystis* ST7 rarely occurs, but in vitro and drug susceptibility studies indicate that this subtype could possess a greater pathogenic potential [13,14,15].

## 2. *Blastocystis* Surface Features

### 2.1. Surface Coat Structure

Different *Blastocystis* isolate exhibit variations of a layer outside the cell membrane called the surface coat. We have hypothesized that this coat could protect the organism from innate host immune response as well as contribute to greater adhesion during colonization [13,16]. One of the earliest studies on the ultrastructure of *Blastocystis* describing a surface coat structure was made by Tan and Zierdt [17]. Using cultured cells, they have identified ameba, granular, and vacuolated forms of the organism. The ameba form featured a filamentous layer as the outermost structure without a distinct cell or membrane. On the other hand, both granular and vacuolated forms had distinct membranes and their filamentous layers appeared more compact and thinner compared with the ameba form. They have observed that it did not have a distinct way of attachment to the cell’s cytoplasm. They have also observed pockets along the layer of the surface coat. Matsumuto et al., using isolates from stool samples and cultures, did not report differences in this filamentous layer. Using a transmission electron microscope, however, they identified a capsule that is composed of a filamentous layer outside the cell membrane [18]. Dunn et al. used *Blastocystis* stocks in cultures and found variations in surface coat structure. There was no apparent association between thickness of the surface coat and median size of the cell [19]. Two cells featured thin surface coats and one of them has the amebic form. Stenzel et al. found that in isolates obtained from colonoscopy samples, there was no distinguishable surface coat. Stocks from cultures, however, showed cells with thick surface coats [20]. Cassidy et al. further confirmed the variation in surface coat structure among several isolates found in different animal hosts [21]. In some cells from cultures, this structure was even completely absent. Although in general, they appeared to be composed of loose fibrils. These studies were done before genotyping isolates became the norm. It is thus difficult to assign morphological characteristics to specific *Blastocystis* STs. 

One of the few and earliest studies on the biochemical properties of *Blastocystis* surface coat used periodic acid-based treatment to detect carbohydrates [22]. This study then positively detected carbohydrates on the *Blastocystis*’ filamentous layer, as well as organelles such as the central vacuole, vesicles, and Golgi apparatus. Lanuza et al. then used lectin probes to identify specific carbohydrates on the surface coat of *Blastocystis* [23]. They detected α-d-mannose, α-d-glucose, *N*-acetyl-α-d-glucosamine (GlcNAc), α-l-fucose, chitin, and sialic acid contained in this structure. Interestingly, some of these are also found on the surfaces of parasitic protozoa as glycoconjugates [24,25]. These components function more than just an additional barrier to the cell membrane. They also play roles in host adhesion and invasion, as well as evasion of host immune response [26,27]. For example, GlcNAc polymers of chitin found in trichomonads are believed to be essential for adherence of the parasite to host cell lectins. In *Leishmania*, mannose and fucose residues found in the parasite’s surface enable the promastigotes to gain entry into the host’s phagocytes [24].

### 2.2. Surface Coat Variations

Our own studies support the notion of variation of morphological forms of *Blastocystis*, including the properties of their surface coats. As we used axenic cultures of identified subtype, we were able assign description to various isolates with accuracy. We used imaging flow cytometry to validate reported morphological forms of *Blastocystis* [9]. We showed that, for example, majority of irregularly-shaped cells, including so-called amoebic forms, are dying cells as indicated by positive propidium-iodide (PI) staining. This was always the case in an ST1 isolate, but less so in ST4 and ST7 isolates. 

**Figure 1:**
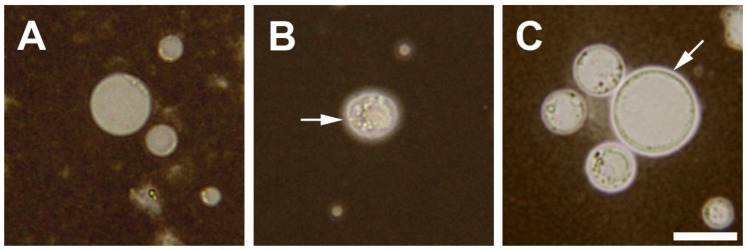
Brightfield microscopy images of India ink-stained *Blastocystis* cells. *Blastocystis* subtype (ST)1 cells (**A**) do not have discernible surface coats, while ST4 (**B**) and ST7 (**C**) isolates have surface coats (arrows) with varied thickness. Cells were prepared from axenic *Blastocystis* cultures in Iscove’s Modified Dulbecco Medium (IMDM) (Life Technologies, Auckland, New Zealand) supplemented with 10% horse serum (Gibco, Auckland, New Zealand)) and stained with India Ink (VWR, Singapore, Singapore). Photomicrographs were taken using BX43 microscope with DP27 camera attachment (Olympus, Tokyo, Japan). Scale bar: 10 μm.

In a study on the susceptibility of *Blastocystis* to a colonic antimicrobial peptide (AMP) LL-37, we also hypothesized that the parasite’s surface coat could act as a barrier preventing this AMP from reaching the membrane. The AMP then will not be able to exert its pore-forming effect on the organism [16]. Indeed, using fluorescent-labeled antibodies against LL-37, we found that *Blastocystis* ST7-B isolate could soak this AMP into its surface, affecting its viability to a lesser degree compared with an ST4-WR1 isolate. Using light microscopy and a negative stain (India ink), we observed that the most susceptible among *Blastocystis* isolates (ST1) have an imperceptible surface coat, while ST7-B isolate’s coat had the thickest and was most dense (Figure 1). 

**Figure 2:**
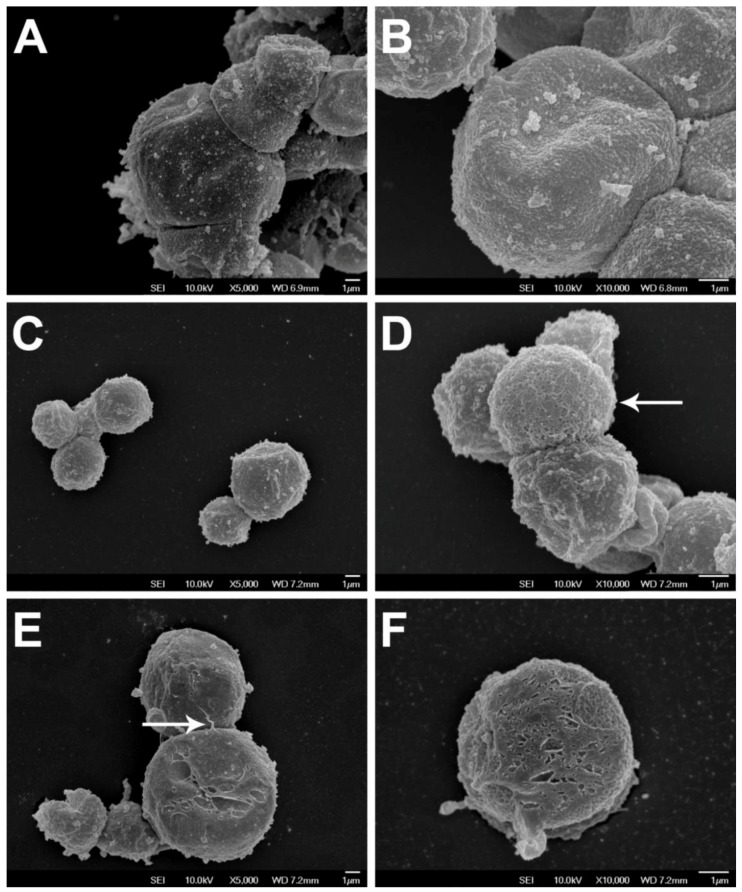
Scanning electron micrographs (SEM) showing *Blastocystis* cells: (**A**,**B**) ST1, (**C**,**D**) ST4, and (**E**,**F**) ST7 isolates. Some cells of *Blastocystis* ST4 (**D**) exhibit a mesh-like appearance in its surface (arrow) and ST7 cells (**E**) appear to have a layer that is continuous with adjacent cells (arrow). Cells were fixed overnight with 4% glutaraldehyde in phosphate-buffered saline solution and attached to 0.1% poly-L-lysine-treated coverslips. After drying and gold-coating, the cells were observed under JSM-6701F scanning electron microscope (JEOL, Tokyo, Japan). Scale bar: 1 μm.

We also obtained scanning electron micrographs (SEM’s) to analyze the surface coats of the three axenized *Blastocystis* isolates. The method and preparation were done as described previously [16]. *Blastocystis* ST1 surface appears more uniform compared with the other isolates (Figure 2A,B), while the surface of ST4 cells were uneven in some areas of the cells (Figure 2C,D). In addition, some cells in ST4 cultures exhibited a mesh-like surface (Figure 2D). *Blastocystis* ST7 cells (Figure 2E,F) appear to have a continuous surface layer connected to adjacent cells (Figure 2E). This is possibly the same material that makes the ST7 cells adhere to each other and contributes to its survival. In an earlier study by Tan et al. [28], SEM’s of ST7 grown as colonies on agar revealed extensive mesh-like connections between the surface coats of cells, supporting the notion that this structure has cytoadherent properties. This is also a plausible explanation for why the cells form a dense mass in cultures and are more difficult to homogenize when in suspension.

### 2.3. Role of Surface Coat

An early study characterizing monoclonal antibodies (MAbs) against *Blastocystis* ST7 identified, by immunogold labelling and biochemical assays, MAbs specific for surface coat carbohydrates and the plasma membrane [29]. Interestingly, the plasma membrane specific MAb (MAb 1D5) was cytotoxic to the parasite, while MAbs against surface carbohydrates were not. MAb 1D5 was subsequently cloned, expressed, and identified as a legumain orthologue [30]. Specific inhibition of *Blastocystis* legumain resulted in programmed cell death features, suggesting a pro-survival role of the enzyme in *Blastocystis*. The data suggested that the surface coat serves to protect essential surface antigens from the host immune system. This helps the parasite survive by not allowing antibodies to be mounted against its vital membrane protein structures [29]. A recent study also indicated that *Blastocystis*’ laterally-acquired genes may have roles in host immune evasion. These genes are associated with host carbohydrates recycling and make it possible for the parasite to express glycosylated molecules on their surface. By covering their surface with host molecules, *Blastocystis* could then escape detection from the host immune system. The authors specifically mentioned, for example, that a gene encoding a β-1,3-galactosyltransferase was laterally acquired by *Blastocystis* from animals. This enzyme functions in the biosynthesis of blood antigens in humans. As mentioned, this could serve to conceal *Blastocystis* from host immune cells by recycling and expressing host molecules on the parasite’s surface [31]. 

## 3. Conclusions

Compared with other intestinal protistan parasites, knowledge on *Blastocystis* biology is still limited. One reason may be that the organism has not been established to be truly pathogenic. This may be a result of the lack of subtype identity in many past studies. It may be that there are truly pathogenic *Blastocystis* STs, while the others can be considered commensals. There is thus a need to expand our knowledge on the specific differences between *Blastocystis* STs. One such difference could lie in the structure of surface coats of the organism. In other protistan parasites, specific surface molecules have already been identified that aid these parasites’ survival [24]. *Blastocystis* may have similar structures in its surface and demonstrating these could help elucidate the pathogenic potential of the parasite. It could also be interesting to determine the role of the surface coat on *Blastocystis*’ nutrition. A few SEM studies on *Blastocystis* have indicated the surface coat to be associated with bacteria [21,32]. It is interesting to know if the parasite could source bacteria for its nutrition by the use of the surface coat, and if so, determine the impact of this process on gut microbiota. Lastly, it may also be worth studying if the surface coat provides additional protection against environmental pressures. It could be that *Blastocystis* STs with thicker surface coats have more resilience against osmotic pressure, extreme pH, and oxygen exposure. Overall, the surface coat is intriguing but poorly studied in *Blastocystis*. Additional knowledge on this structure would contribute significantly to *Blastocystis* biology.
